# Strategies to prevent surgical site infections in acute-care hospitals: 2022 Update

**DOI:** 10.1017/ice.2023.67

**Published:** 2023-05-04

**Authors:** Michael S. Calderwood, Deverick J. Anderson, Dale W. Bratzler, E. Patchen Dellinger, Sylvia Garcia-Houchins, Lisa L. Maragakis, Ann-Christine Nyquist, Kiran M. Perkins, Michael Anne Preas, Lisa Saiman, Joshua K. Schaffzin, Marin Schweizer, Deborah S. Yokoe, Keith S. Kaye

**Affiliations:** 1 Dartmouth Hitchcock Medical Center, Lebanon, New Hampshire, United States; 2 Duke Center for Antimicrobial Stewardship and Infection Prevention, Duke University School of Medicine, Durham, North Carolina, United States; 3 University of Oklahoma Health Sciences Center, Oklahoma City, Oklahoma, United States; 4 University of Washington Medical Center, Seattle, Washington, United States; 5 The Joint Commission, Oakbrook Terrace, Illinois, United States; 6 Johns Hopkins School of Medicine, Baltimore, Maryland, United States; 7 Children’s Hospital Colorado, University of Colorado School of Medicine, Aurora, Colorado, United States; 8 Division of Healthcare Quality Promotion, Centers for Disease Control and Prevention, Atlanta, Georgia, United States; 9 University of Maryland Medical System, Baltimore, Maryland, United States; 10 Columbia University Irving Medical Center and NewYork–Presbyterian Hospital, New York, New York, United States; 11 Children’s Hospital of Eastern Ontario, University of Ottawa, Ottawa, Ontario, Canada; 12 Center for Access and Delivery Research and Evaluation, Iowa City VA Health Care System, University of Iowa, Iowa City, Iowa; 13 University of California-San Francisco, San Francisco, California, United States; 14 Rutgers Robert Wood Johnson Medical School, New Brunswick, New Jersey, United States

## Abstract

The intent of this document is to highlight practical recommendations in a concise format designed to assist acute-care hospitals in implementing and prioritizing their surgical site infection (SSI) prevention efforts. This document updates the *Strategies to Prevent Surgical Site Infections in Acute Care Hospitals* published in 2014.^1^ This expert guidance document is sponsored by the Society for Healthcare Epidemiology of America (SHEA). It is the product of a collaborative effort led by SHEA, the Infectious Diseases Society of America (IDSA), the Association for Professionals in Infection Control and Epidemiology (APIC), the American Hospital Association (AHA), and The Joint Commission, with major contributions from representatives of a number of organizations and societies with content expertise.

## Summary of major changes

This section lists major changes from the *Strategies to Prevent Surgical Site Infections in Acute Care Hospitals: 2014 Update*,^1^ including recommendations that have been added, removed, or altered. Recommendations are categorized as essential practices that should be adopted by all acute-care hospitals (in 2014 these were “basic practices,” renamed to highlight their importance as a foundation for hospitals’ healthcare-associated infection (HAI) prevention programs) or additional approaches that can be considered for use in locations and/or populations within hospitals when SSIs are not controlled after implementation of essential practices (in 2014 these were called “special approaches”). See Table 1 for a complete summary of recommendations contained in this document.

### Essential practices

- Modified recommendation to administer prophylaxis according to evidence-based standards and guidelines to emphasize that antimicrobial prophylaxis should be discontinued at the time of surgical closure in the operating room.
- The use of parenteral and oral antibiotics prior to elective colorectal surgery is now considered an essential practice. This recommendation was included in the 2014 document but was a sub-bullet recommendation. This recommendation was elevated to its own recommendation for increased emphasis.
- Reclassified decolonization of surgical patients with an anti-staphylococcal agent for cardiothoracic and orthopedic procedures from an Additional Approach to an Essential Practice.
- The use of vaginal preparation with an antiseptic solution prior to cesarean delivery and hysterectomy was added as an essential practice.
- Reclassified intraoperative antiseptic wound lavage from an Additional Approach to an Essential Practice. However, this approach should only be used when sterility of the antiseptic can be ensured and maintained.
- Control of blood-glucose levels during the immediate postoperative period for all patients was modified (1) to emphasize the importance of this intervention regardless of a known diagnosis of diabetes mellitus, (2) to elevate the evidence level to “high” for all procedures, and (3) to lower the target glucose level from <180 mg/dL to 110–150 mg/dL.
- Reclassified use of bundles to promote adherence with best practices from Unresolved to an Essential Practice. Discussion of the use of checklists and bundles was combined for this recommendation.
- Reclassified observe and review operating room personnel and the environment of care in the operating room and central sterile reprocessing from an Additional Approach to an Essential Practice.

### Additional approaches

- Reclassified the recommendation to perform an SSI risk assessment from an Essential Practice to an Additional Approach.
- The use of negative pressure dressings was added as an Additional Practice. To date, available evidence suggests that this strategy is most likely effective in specific procedures (eg, abdominal procedures) and/or specific patients (eg, increased body mass index).
- Reclassified the use of antiseptic-impregnated sutures from Not Recommended to Additional Approaches.

### Not recommended

- Expanded discussion on the recommendation against the routine use of vancomycin for antimicrobial prophylaxis.

### Unresolved issues

- Reclassified the use of supplemental oxygen for patients requiring mechanical ventilation from an Essential Practice to Unresolved.
- Added discussion on the use of antimicrobial powder.
- Added discussion on the use of surgical attire as a strategy to prevent SSI.

## Intended Use

This document was developed following the process outlined in the *Handbook for SHEA-Sponsored Guidelines and Expert Guidance Documents*.^2^ No guideline or expert guidance document can anticipate all clinical situations, and this document is not meant to be a substitute for individual clinical judgment by qualified professionals.

This document is based on a synthesis of evidence, theoretical rationale, current practices, practical considerations, writing-group consensus, and consideration of potential harm, when applicable. A summary list of recommendations is provided along with the relevant rationale in Table 1.

## Methods

SHEA recruited 3 subject-matter experts in the prevention of SSI to lead the panel of members representing the Compendium partnering organizations—SHEA, IDSA, APIC, AHA, and The Joint Commission, as well as representation by the Centers for Disease Control and Prevention (CDC).

SHEA utilized a consultant medical librarian, who developed a comprehensive search strategy for PubMed and Embase (January 2012–July 2019, updated to August 2021). Article abstracts were reviewed by panel members. Each abstract was reviewed by at least 2 reviewers using the abstract management software Covidence (Melbourne, Australia), and selected abstracts were reviewed as full text. In July 2021, the Compendium Lead Authors group voted to update the literature findings, and the librarian re-ran the search to update it to August 2021. Panel members reviewed the search yield via Covidence and incorporated relevant references.

Recommendations resulting from this literature review process were classified based on the quality of evidence and the balance between desirable and potential for undesirable effects of various interventions (Table 2). Panel members met via video conference to discuss literature findings; recommendations; quality of evidence for these recommendations; and classification as essential practices, additional practices, or unresolved issues. Panel members reviewed and approved the document and its recommendations.

The Compendium Expert Panel, made up of members with broad healthcare epidemiology, surgical, and infection prevention expertise, reviewed the draft manuscript after consensus had been reached by writing-panel members.

Following review and approval by the Expert Panel, the 5 Compendium partners, collaborating professional organizations, and CDC reviewed the document. Prior to dissemination, the guidance document was reviewed and approved by the SHEA Guidelines Committee, the IDSA Practice Standards and Guidelines Committee, AHA, and The Joint Commission, and the Boards of SHEA, IDSA, and APIC.

All panel members complied with the SHEA and IDSA policies on conflict-of-interest disclosure.

## Section 1: Rationale and statements of concern

### Burden of outcomes associated with SSI

1. Surgical site infections (SSIs) are common complications in acute-care facilities.
  - SSIs occur in ~1%–3% of patients undergoing inpatient surgery, depending on the type of operative procedure performed.^3,4^ In total, 21,186 SSIs were reported to the CDC National Healthcare Safety Network (NHSN) in 2021 from a total of 2,759,027 operative procedures.^3^
  - Additional data on ambulatory and outpatient surgeries are needed. Overall, many of these procedures are lower risk by virtue of procedure type and patient selection, and some may involve minimally invasive techniques that have a lower risk of infection.^5,6^ It is important to mention, however, that both inpatient and ambulatory operating rooms need to adhere to strict infection prevention standards.
  - SSIs now are one of the most common and most costly HAIs.^7-11^
2. Up to 60% of SSIs are preventable using evidence-based guidelines.^12,13^
3. When not prevented, SSIs can result in a significant increase in postoperative hospital days and many also require reoperation, both during the initial surgical admission and during hospital readmission.^11,14-16^
4. Patients with an SSI have a 2–11 times higher risk of death compared to operative patients without SSI.^17,18^ Also, 77% of deaths in patients with SSI are directly attributable to SSI.^19^
5. Attributable costs of SSI vary depending on the type of operative procedure, medical implants, and the type of infecting pathogen.^16,18,20-27^ Overall, it is estimated that the cost of care for patients who develop an SSI is 1.4–3 times higher than for patients who do not develop an SSI.^28^ Deep-incisional and organ-space SSIs are associated with the highest cost.^28^ All studies evaluated in a systematic review reported some economic benefit associated with SSI prevention, but there is significant heterogeneity in the literature related to cost accounting.^29,30^ In the United States, SSIs are believed to account for $3.5 billion to $10 billion annually in healthcare expenditures.^31,32^
6. Finally, data reported to the CDC NHSN show that SSIs can be caused by antibiotic-resistant bacteria such as methicillin-resistant *Staphylococcus aureus*, vancomycin-resistant Enterococci, and multidrug-resistant gram-negative bacilli. These infections can be more difficult to manage and can be caused by pathogens that are resistant to standard empiric antibiotics.^33^

### Risk factors for SSI

1. Numerous risk factors have been described for SSI, including intrinsic factors, patient-specific risk factors, and perioperative factors related to surgical practices (Table 3). Some common patient-specific risk factors include obesity, diabetes, immuno-suppressive therapy, malnutrition, and smoking. In pediatrics, premature infants are also at higher risk, especially those undergoing gastrointestinal surgery early in life. Examples of perioperative risk factors include inadequacies in surgical scrub, the antiseptic preparation of the skin, antimicrobial prophylaxis, and duration of surgery.
2. The CDC NHSN–determined risk factors for different procedure categories are incorporated in the calculation of the standardized infection ratio (SIR).^34^

## Section 2: Background on detection of SSI

### Surveillance definitions for SSI

**Fig. 1.**
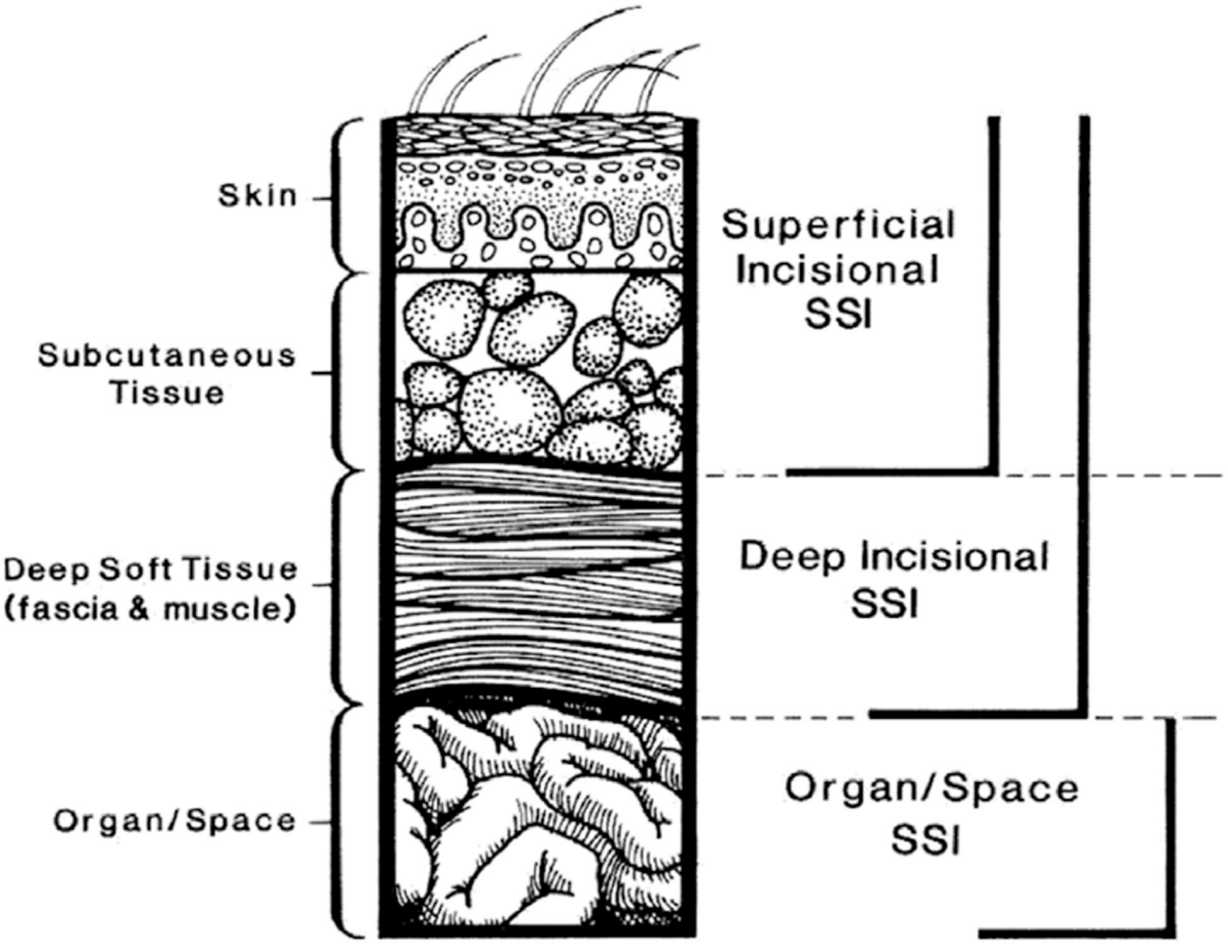
CDC National Healthcare Safety Network (NHSN) classification for surgical site infection. Modified from Horan TC, et al.^362^ CDC definitions of nosocomial surgical site infections, 1992.

1. Surveillance definitions must be established and consistently applied over time to make comparisons within and between institutions meaningful.
  - NHSN definitions for SSI are widely used for public reporting, interfacility comparison, and pay-for-performance comparisons,^35-38^ based on selected procedures identified by procedure codes assigned from the *International Classification of Diseases, 10^th^ Revision* Clinical Modifications/Procedure Coding System (ICD-10-CM/PCS) and/or current procedural terminology (CPT) codes.^35-37^
  - Validation of the application of surveillance definitions between data abstractors may be necessary to ensure consistent application.^41,42^
2. According to widely used CDC NHSN definitions,^43^ SSIs are classified as follows (Fig. 1):
  - Superficial incisional (involving only skin or subcutaneous tissue of the incision)
    - Superficial incisional primary (SIP): SSI identified in a primary incision in a patient with 1 or more incisions.
    - Superficial incisional secondary (SIS): SSI identified in the secondary incision in a patient that has had an operation with >1 incision.
  - Deep incisional (involving fascia and/or muscular layers)
    - Deep-incisional primary (DIP): SSI identified in a primary incision in a patient who has had an operation with 1 or more incisions.
    - Deep-incisional secondary (DIS): SSI identified in a secondary incision in a patient who has had an operation with > 1 incision.
  - Organ-space: Involving any part of the body opened or manipulated during the procedure, excluding skin incision, fascia, or muscle layers.

### Surveillance methods for SSI and detection of patients

1. The most accurate method of SSI surveillance is the direct method for case finding with daily observation of the surgical site by a physician, advanced practice provider, registered nurse, or infection preventionist starting 24–48 hours postoperatively.^15,44-46^ Although the direct method of case finding has been used as the “gold standard” for some studies, it is rarely used by infection prevention staff due to its high resource utilization requirements and impracticality.
2. The indirect method of case finding is less time-consuming than the direct method; it can be performed using criteria or algorithms applied to electronic records; and it can be performed retrospectively.
  - The indirect method of case finding consists of 1 or a combination of the following as appropriate based on inpatient or outpatient surveillance and the setting:
    - Review of microbiology reports and patient medical records
    - Surgeon and/or patient surveys by mail, telephone, or web-based application^47^
    - Patient or family interview, particularly when postoperative care is remote and/or follow-up care is being provided by an alternative provider
    - Screening for early or additional postoperative visits, readmission, and/or return to the operating room
    - Other information such as coded diagnoses, coded procedures, operative reports, or antimicrobials ordered
  - Indirect methods of SSI surveillance have been demonstrated to be reliable (sensitivity, 84%–89%) and specific (specificity, 99.8%) compared to the “gold standard” of direct surveillance.^48-50^ Components of the indirect methods that were associated with highest sensitivities included review of nursing notes, billing codes, and antimicrobials used.
  - Indirect methods for SSI surveillance are less reliable for surveillance of superficial-incisional infections, particularly those occurring after discharge.^51^
3. Automated data systems and electronic health records should be used to improve efficiency, improve sensitivity, and broaden SSI surveillance.^50^
  - SSI surveillance can be expanded by utilizing hospital databases that include administrative claims data (including diagnosis and procedure codes), antimicrobial days, readmission to the hospital, return to the operating room and/or by implementing a system that imports automated microbiologic culture data, surgical procedure data, and general demographic information into a single surveillance database.^52-54^
  - These methods improve the sensitivity of indirect surveillance for detection of SSI and reduce the effort of the infection preventionist.^52^
  - Medicare claims data can be used to enhance surveillance methods for SSI and to identify hospitals with unusually high or low rates of SSI.^55,56^
  - Administrative data can be used to increase the efficiency of SSI reporting and validation.^57-59^
  - Use of algorithms,^58^ machine learning,^60^ and predictive models may be helpful in surveillance of SSIs.
  - Administrative and automated data used for surveillance purposes should be validated to ensure accuracy.
  - Electronic health record (EHR) vendors should increase standardization and automated collection of key metrics. The focus should be to reduce data burden on hospital and health-system staff.
4. The proportion of SSIs detected through postdischarge surveillance can vary by surveillance method, operative setting, type of SSI, and surgical procedure.
  - The majority of surgical procedures are now outpatient procedures.^61^ In addition, length of stay following inpatient procedures has decreased. Surveillance methodologies must take these practice changes into account.
  - Superficial incisional SSIs are most commonly detected and managed in the outpatient setting. In contrast, deep-incisional and organ-space infections typically require readmission to the hospital for management.^51^
  - Surveillance for SSIs in the ambulatory care setting is challenging because patients may not return to the same organization for routine postoperative care^62^ or for management of complications.^63^
5. CDC is prescriptive about denominator data collection^43^; however, it is less prescriptive on how possible cases (numerator data) should be identified for evaluation.
  - Differences in case finding methodology may lead to variability in surveillance rates.^64^
  - CDC encourages standardization of data sources for more consistent reporting. Both state health departments and the CMS select hospitals for data validation.
  - By improving completeness of reporting, the overall institutional SSI rate typically increases.^65-67^ As more data sources are used, the detection of SSIs is likely to increase.^52^

## Section 3: Background on prevention of SSI

### Summary of existing guidelines, recommendations, and requirements

A number of guidelines are available on the prevention of SSIs, and our writing panel compared and contrasted some of the differences in developing our current recommendations.^68^ We list some of these guidelines below, along with current US reporting requirements.

1. CDC and Healthcare Infection Control Practices Advisory Committee (HICPAC) guidelines^4,69^
2. American College of Surgeons and Surgical Infection Society SSI Guidelines^70^
3. World Health Organization 2018^71^
4. National Institute for Health and Clinical Excellence (NICE)—United Kingdom 2008^57,58^
5. SHEA Expert Guidance: Infection Prevention in the Operating Room Anesthesia Work Area^72^
6. American Society of Health-System Pharmacists (ASHP) Clinical Practice Guideline for Antimicrobial Prophylaxis in Surgery 2013^73^
7. Institute for Healthcare Improvement (IHI)^74^
  - The IHI created a nationwide quality improvement project to improve outcomes in hospitalized patients,^75,76^ including 6 preventive measures for SSI that are also included in the 100,000 and 5 Million Lives Campaigns.^75,76^
8. Federal requirements
  - Centers for Medicare & Medicaid Services (CMS)
    - In accordance with the Deficit Reduction Act of 2005, US hospitals that are paid by Medicare under the acute-care inpatient prospective payment system receive their full Medicare Annual Payment Update only if they submit required quality measure information to CMS.
    - In addition, US acute-care hospitals submit data to the NHSN for complex SSIs following colon surgery and abdominal hysterectomy. These data are publicly reported on the CMS Hospital Care Compare website^77,78^ and are used to determine pay-for-performance in both the Hospital-Acquired Condition Reduction Program^79^ and the Hospital-Value Based Purchasing Program.^80^
    - Accrediting organizations with deeming authority granted by the CMS, such as The Joint Commission and Det Norske Veritas Healthcare (DNV), verify that CMS requirements are met as part of the accreditation process.

### Infrastructure requirements

Facilities performing surgery should have the following elements in place:

1. Trained infection prevention personnel
  - Infection preventionists (1) must be specifically trained in methods of SSI surveillance, (2) must have knowledge of and the ability to prospectively apply the CDC/NHSN definitions for SSIs, (3) must possess basic computer and mathematical skills, and (4) must be adept at providing feedback and education to healthcare personnel (HCP) when appropriate.^4,81^
  - Having an increased number of infection preventionists, certified infection preventionists, and a hospital epidemiologist are associated with lower rates of SSI. A specific threshold for staffing has not been defined.^82^
2. Education for HCP
  - A surgeon leader or champion can be a critical partner in changing culture and improving adherence to prevention practices.
  - Regularly provide education to surgeons and perioperative personnel through continuing education activities directed at minimizing perioperative SSI risk through implementation of recommended process measures.
    - Combine several educational components into concise, efficient, and effective recommendations that are easily understood and remembered.^83^
    - Provide education regarding the outcomes associated with SSI, risks for SSI, and methods to reduce risk to all surgeons, anesthesiologists, and perioperative personnel.
  - Ensure that education and feedback regarding SSI rates and specific measures that can be used to prevent infection filter down to all frontline multidisciplinary HCPs providing care in the perioperative^84^ and postoperative settings.^85^
3. Education of patients and families. Provide education for patients and patients’ families to reduce risk associated with intrinsic patient-related SSI risk factors.^86,87^
4. Computer-assisted decision support and automated reminders
  - Several institutions have successfully employed computer-assisted decision support methodology to improve the rate of appropriate administration of antimicrobial prophylaxis (including re-dosing during prolonged cases).^88-91^
  - Computer-assisted decision support can be time-consuming to implement,^72^ and institutions must appropriately validate computer-assisted decision support systems after implementation to ensure that they are functioning appropriately.^92^
5. Utilization of automated data
  - Install information technology infrastructure to facilitate data transfer, receipt, and organization to aid with tracking of process and outcome measures.
  - Consider use of data mining software to identify potential SSIs which can then be further evaluated.
  - Consider leveraging existing electronic health record capabilities to provide process measure information that informs improvement approaches.

## Section 4: Recommended strategies to prevent SSI

Recommendations are categorized as either (1) essential practices that should be adopted by all acute-care hospitals or (2) additional approaches that can be considered when hospitals have successfully implemented essential practices and seek to further improve outcomes in specific locations and/or patient populations. Essential practices include recommendations in which the potential to affect HAI risk clearly outweighs the potential for undesirable effects. Additional approaches include recommendations in which the intervention is likely to reduce HAI risk but there is concern about the risks for undesirable outcomes, recommendations for which the quality of evidence is low, or recommendations where the evidence supports the effect of the intervention in select settings (e.g., during outbreaks) or for select patient populations. Hospitals can prioritize their efforts by initially implementing infection prevention approaches listed as essential practices. If HAI surveillance or other risk assessments suggest that there are ongoing opportunities for improvement, hospitals should consider adopting some or all of the infection prevention approaches listed as additional approaches. These approaches can be implemented in specific locations or patient populations or can be implemented hospital-wide, depending on outcome data, risk assessment, and/or local requirements. Each infection prevention recommendation is given a quality of evidence grade (Table 2).

### Essential practices for preventing SSI recommended for all acute-care hospitals

1. **Administer antimicrobial prophylaxis according to evidence-based standards and guidelines.**^75^ (Quality of evidence: HIGH)
  - Begin administration within 1 hour prior to incision to maximize tissue concentration.^73,93,94^ Administering an antimicrobial agent <1 hour prior to incision is effective; some studies show superior efficacy for administration between 0 and 30 minutes prior to incision compared with administration between 30 and 60 minutes prior to incision.^95,96^
    - Two hours are allowed for the administration of vancomycin and fluoroquinolones due to longer infusion times.
    - For cesarean delivery, administer antimicrobial prophylaxis prior to skin incision rather than after cord clamping.^97^
    - In procedures using “bloodless” techniques, many experts believe that antimicrobial agents should be infused prior to tourniquet inflation, though data are lacking to inform this recommendation.^98^
  - Select appropriate antimicrobial agents based on the surgical procedure, the most common pathogens known to cause SSI for the specific procedure, and published recommendations.^73^
    - Although it is not recommended to routinely use vancomycin, this agent should be considered in patients who are known to be MRSA colonized (including those identified on preoperative screening), particularly if the surgery involves prosthetic material.
  - Obtain a thorough allergy history. Self-reported β-lactam allergy has been linked to a higher risk of SSI due to use of alternative, non–β-lactam and often inferior antibiotics, and many patients with a self-reported β-lactam allergy can safely receive a β-lactam antibiotic as prophylaxis.^99-101^
  - Discontinue antimicrobial agents after incisional closure in the operating room.^73^
    - Although some guidelines suggest stopping the antimicrobial agents within 24 hours of surgery, there is no evidence that antimicrobial agents given after incisional closure contribute to reduced SSIs^102^ even when drains are inserted during the procedure.^103^ In contrast, antibiotics given after closure contribute to increased antimicrobial resistance^104,105^ and increased risk of *Clostridioides difficile* infection^106^ and acute kidney injury.^107^
    - In a single-center, retrospective, cohort study comparing joint arthroplasty, patients who received a single dose of antibiotic prophylaxis (no additional doses after skin closure) versus 24-hour antibiotic administration, there were no differences in the following outcomes between these 2 groups: prosthetic joint infection, superficial infection, 90-day reoperation, and 90-day complications.^108^
  - Adjust dosing based on patient weight,^73^ according to the following examples:
    - For cefazolin, use 30–40 mg/kg for pediatric patients, use 2 grams for patients weighing ≤120 kg, and 3 grams for patients weighing >120 kg.^109,110^ Although data are conflicting regarding the role of 3 grams of cefazolin dosing in reducing SSI in obese patients, multiple studies have shown a benefit compared to 2-gram dosing in this patient population,^110-112^ with few adverse events from a single dose of 3 grams versus 2 grams of cefazolin. Although some hospitals use 1 gram for adult patients weighing ≤80 kg, there is no harm associated with giving a 2-gram dose.
    - Dose vancomycin at 15 mg/kg.^113^
    - Dose gentamicin at 5 mg/kg for adult patients and 2.5 mg/kg for pediatric patients. For morbidly obese patients receiving gentamicin, use the ideal weight plus 40% of the excess weight for dose calculation.^114^
  - Re-dose prophylactic antimicrobial agents for lengthy procedures and in cases with excessive blood loss during the procedure (ie, >1,500 mL).^73^ Re-dose prophylactic antimicrobial agents at intervals of 2 half-lives (measured from the time the preoperative dose was administered) in cases that exceed this period. For example, re-dose cefazolin after 4 hours in procedures >4 hours long.^73^
2. **Use a combination of parenteral and oral antimicrobial prophylaxis prior to elective colorectal surgery to reduce the risk of SSI.**^115,116^ (Quality of evidence: HIGH)
  - A 2019 meta-analysis of 40 studies (28 randomized clinical trials [RCTs] and 12 observational studies) found that the combination of parenteral and oral antimicrobial prophylaxis and mechanical bowel preparation prior to elective colorectal surgery significantly reduces SSI, postoperative ileus, anastomotic leak, and 30-day mortality, without an increase in *C. difficile* infection.^116^ In 2021,^117^ the meta-analysis was updated to include the results from the MOBILE and ORALEV trials, which further demonstrated the decreases shown in 2019,^119,120^ along with data showing that oral antimicrobial prophylaxis alone without mechanical bowel preparation significantly reduces SSI, anastomotic leak, and 30-day mortality.^121,122^ We continue to recommend the combination of parenteral and oral antimicrobial prophylaxis and mechanical bowel preparation prior to elective colorectal surgery, unless there is a contraindication to mechanical bowel preparation, in which case, only parenteral and oral antimicrobial prophylaxis should be administered.
  - Use of combination parenteral and oral antimicrobial agents to reduce the risk of SSI should be considered in any surgical procedure where entry into the colon is possible or likely, as in gynecologic oncology surgery.
  - Mechanical bowel preparation without use of oral antimicrobial agents does not decrease the risk of SSI.^115^ A recent prospective randomized multicenter trial confirmed earlier meta-analysis findings, with significantly higher SSI and anastomotic leakage in patients who received mechanical bowel preparation without oral antimicrobial agents.^122^
3. **Decolonize surgical patients with an antistaphylococcal agent in the preoperative setting for orthopedic and cardiothoracic procedures.** (Quality of evidence: HIGH). **Decolonize surgical patients for other procedures at high risk of staphylococcal SSI, such as those involving prosthetic material.** (Quality of evidence: LOW)
  - Decolonization refers to the practice of treating patients with an antimicrobial and/or antiseptic agent to suppress *S. aureus* colonization inclusive of both methicillin-susceptible *S. aureus* (MSSA) and methicillin-resistant *S. aureus* (MRSA).
    - Published data are most supportive of using intranasal mupirocin and chlorhexidine bathing. There are some preliminary data on intranasal povidone-iodine administered immediately before surgery. This approach may have practical advantages, but more data are needed.^124^ Fewer data exist for other alternative strategies such as intranasal alcohol-based antisepsis and phototherapy.
    - The strongest data recommend up to 5 days of intranasal mupirocin (twice daily) and bathing with chlorhexidine gluconate (CHG) (daily).
  - A meta-analysis of 17 studies of patients undergoing cardiac or orthopedic procedures concluded that decolonization strategies prevent *S. aureus* SSIs.^125^
  - Some trials demonstrated that preoperative screening for *S. aureus*, combined with intranasal mupirocin and CHG bathing, was effective in reducing SSI.
    - For example, a randomized, double-blind, placebo-controlled, multicenter trial showed that rapid identification of *S. aureus* nasal carriers, followed by decolonization with intranasal mupirocin and CHG bathing was associated with a >2-fold reduction in the risk for postoperative infection due to *S. aureus* and an almost five-fold reduction in incidence of deep-incisional SSI due to *S. aureus*.^126^ Patients undergoing clean procedures (eg, cardiothoracic, orthopedic, vascular) who were randomized to decolonization also had reduced 1-year mortality compared with those patients who were randomized to the placebo.^127^
    - A 20-hospital, nonrandomized, quasi-experimental study of patients undergoing cardiac surgery or total joint arthroplasty found a significant decrease in deep-incisional or organ-space *S. aureus* SSI after implementing a bundle of interventions, including *S. aureus* nasal screening, decolonization of nasal carriers with mupirocin, CHG bathing for all patients, and perioperative antibiotic prophylaxis adjustment based on MRSA carriage status.^128^
    - Notably, universal decolonization for targeted procedures is likely more cost effective than screen-and-treat strategies.^129,130^ Universal decolonization may also be easier to implement.
    - Some hospitals continue to use screen-and-treat strategies because the results from screening for MRSA colonization can guide antibiotic prophylaxis.
  - In contrast, other trials that assessed a wide range of surgical specialties did not observe a protective effect against SSIs.
    - A prospective, interventional, cohort study with crossover design involving 21,000 patients concluded that universal, rapid screening for MRSA at admission combined with decolonization of carriers did not reduce the SSI rate due to MRSA.^131^ This study included 8 surgical specialties: abdominal surgery, orthopedics, urology, neurosurgery, cardiovascular surgery, thoracic surgery, plastic surgery, and solid-organ transplantation. Similarly, a prospective interventional cohort study of 10 hospitals did not find a decrease in MRSA clinical cultures when MRSA screening and decolonization were performed among 9 surgical specialties. However, when the analysis was limited to patients undergoing clean surgery, MRSA screening and decolonization was significantly associated with reductions in MRSA SSI rates.^132,133^ Clean surgery included cardiothoracic, neuro, orthopedic, plastic, and vascular surgery.
    - A double-blinded, randomized-controlled trial involving >4,000 patients undergoing general, gynecologic, neurologic, or cardiothoracic surgery showed that universal intranasal mupirocin application, when not combined with CHG bathing, did not significantly reduce the *S. aureus* SSI rate.^134^ In a secondary analysis of this data, the use of intranasal mupirocin was associated with an overall decreased rate of nosocomial *S. aureus* infections among the *S. aureus* carriers.
  - A Cochrane review concluded that mupirocin decolonization of the nares alone may be effective, particularly in certain groups, including patients undergoing orthopedic and cardiothoracic procedures.^135^ However, routine preoperative decolonization with mupirocin without screening may lead to mupirocin resistance.^136^
  - Routine decolonization with antiseptic agents such as intranasal povidone-iodine without screening can be performed because povidone-iodine resistance has not been observed.
    - One single-center RCT comparing intranasal povidone-iodine with mupirocin in total joint arthroplasty and spinal surgery patients found that povidone-iodine and mupirocin were similarly effective.^137^ In that RCT, topical CHG wipes in combination with povidone-iodine was given within 2 hours of surgery versus with mupirocin during the 5 days before surgery.^137^ There was no significant difference between deep SSI rates when comparing those who received povidone-iodine with those who received mupirocin.
    - Two quasi-experimental, single-center studies of intranasal povidone-iodine decolonization reported a significant reduction in SSIs when compared with standard care among preintervention groups. One study paired intranasal povidone-iodine decolonization with CHG wipes and oral povidone-iodine rinse for elective orthopedic surgery^138^; the other study paired it with CHG wipes or baths and povidone-iodine skin antisepsis for urgent lower extremity repairs of fractures that required hardware.^139^
  - Data are mixed on at-home preoperative bathing with CHG-containing products alone for patients not known to be colonized with *Staphylococcus aureus*.
    - Preoperative bathing with agents such as CHG has been shown to reduce bacterial colonization of the skin.^140,141^ Several studies have examined the utility of preoperative showers, but none has definitively proven that they decrease SSI risk. A Cochrane review evaluated the evidence for preoperative bathing or showering with antiseptics for SSI prevention.^142^ Six RCTs evaluating 4% CHG use were included in the analysis, with no clear evidence of benefit noted. Several of these studies had methodologic limitations and were conducted several years ago. Thus, the role of preoperative bathing in SSI prevention remains uncertain.
    - To achieve the maximum antiseptic effect of CHG, adequate levels of CHG must be achieved and maintained on the skin. Typically, adequate levels are achieved by allowing CHG to dry completely. Additional strategies for preoperative bathing with CHG, such as preimpregnated cloths, have shown promise,^143-145^ but data are currently insufficient to support this approach.
4. **Use antiseptic-containing preoperative vaginal preparation agents for patients undergoing cesarean delivery or hysterectomy.** (Quality of evidence: MODERATE)
  - Use of povidone-iodine or CHG-based vaginal preparation agents immediately before cesarean delivery reduces endometritis by 59%, with possibly even greater benefit among women in labor.^146^ Products should be chosen and used in accordance with manufacturer’s instructions for use.
  - Vaginal preparation with antiseptic solution is also recommended for elective hysterectomy.^147^
5. **Do not remove hair at the operative site unless the presence of hair will interfere with the surgical procedure.**^4,119^ (Quality of evidence: MODERATE)
  - If hair removal is necessary in elective procedures, remove hair outside the operating room using clippers or a depilatory agent.
  - Razors may be acceptable for hair removal in a subset of procedures (eg, procedures involving male genitalia). One small, single-center, RCT demonstrated that clipping hair on the scrotum can cause more skin trauma than razors; clipping hair did not decrease the rate of SSI.^148^
6. **Use alcohol-containing preoperative skin preparatory agents in combination with an antiseptic.** (Quality of evidence: HIGH)
  - Alcohol is highly bactericidal and effective for preoperative skin antisepsis, but it does not have persistent activity when used alone. Rapid, persistent, and cumulative antisepsis can be achieved by combining alcohol with CHG or an iodophor.^149^ Alcohol is contraindicated for certain procedures due to fire risk, including procedures in which the preparatory agent may pool or not dry (eg, involving hair). Alcohol may also be contraindicated for procedures involving mucosa, cornea, or ear.
  - The most effective antiseptic to combine with alcohol remains unclear; however, data from recent trials favor the use of CHG–alcohol over povidone-iodine–alcohol.
    - A Cochrane review of 13 studies, published in 2015, was inconclusive regarding the best strategy for preoperative skin antisepsis.^150^ Only 1 of these studies compared 0.5% CHG–alcohol to povidone-iodine–alcohol.
    - Four RCTs (3 single center and 1 multicenter) have compared CHG–alcohol to povidone-iodine–alcohol.
      - Tuuli et al^151^ conducted a single-center RCT of 1,147 women undergoing cesarean delivery. Women randomized to receive CHG–alcohol had a 45% reduction in SSI compared to women randomized to receive povidone-iodine–alcohol (relative risk, 0.55; 95% confidence interval, 0.34–0.90; *P* = .02).
      - Ritter et al^152^ conducted a single-center RCT of 279 patients undergoing lower-limb procedures. Patients randomized to receive povidone-iodine–alcohol had a 3.5-fold higher rate of wound healing complications, including SSI, compared with patients randomized to receive CHG-alcohol.
      - Broach et al^153^ conducted a single-center, noninferiority RCT of 802 patients undergoing elective, clean-contaminated colorectal procedures. The SSI rate was higher among patients randomized to receive povidone-iodine–alcohol (18.7% vs 15.9%), which failed to meet criterion for noninferiority compared to CHG–alcohol.
      - Charehbili et al^154^ conducted a multicenter, cluster-randomized trial with crossover among 3,665 patients undergoing breast, vascular, colorectal, gallbladder, or orthopedic procedures. No difference in SSI rates was observed between the 2 groups, but some concerns were raised about the methods, including cluster sample size, number of clusters, and how the treatment period was analyzed.^155^
    - CHG–alcohol is the antiseptic of choice for patients with *S. aureus* colonization.^128^
    - In the absence of alcohol, CHG may have advantages over povidone-iodine, including longer residual activity and activity in the presence of blood or serum.^156,157^
    - Antiseptics are not interchangeable. Follow manufacturer’s instructions to ensure correct application. Topical CHG preparations may be contraindicated for use in mouth, eyes and ears, patients with skin disease involving more than the superficial layers of skin, and procedures involving the meninges. Use of topical CHG preparations for preterm infants is controversial due to concerns for skin toxicity, absorption, and resultant toxicity including neurotoxicity.^158^ However, apart from these specific contraindications, topical CHG for skin antisepsis and SSI prevention has been shown to be safe.^158-162^
7. **For procedures not requiring hypothermia, maintain normothermia (temperature >35.5°C) during the perioperative period.** (Quality of evidence: HIGH)
  - Even mild hypothermia can increase SSI rates. Hypothermia may directly impair neutrophil function or impair it indirectly by triggering subcutaneous vasoconstriction and subsequent tissue hypoxia. Hypothermia may increase blood loss, leading to wound hematomas or the need for transfusion—both of which can increase SSI rates.^163^
  - RCTs have shown the benefits of both preoperative and intraoperative warming in reducing SSI rates and intraoperative blood loss.^164-166^
  - Preoperative normothermia may be most beneficial^167^; patients who received 30 minutes of preoperative warming had lower intraoperative hypothermia rates.^168^ One study used 2 hours of preoperative warming, but a meta-analysis suggested that 30 minutes should be sufficient.
  - Patients who are hypothermic at the end of surgery may remain hypothermic for up to 5 hours. Although there is not a standardized duration of postoperative warming, one study used 2 hours of postoperative warming and showed reduced rates of SSI.
8. **Use impervious plastic wound protectors for gastrointestinal and biliary tract surgery.** (Quality of evidence: HIGH)
  - A wound protector, a plastic sheath that lines a wound, facilitates retraction of an incision during surgery without the need for additional mechanical retractors.
  - A recent meta-analysis of 14 randomized clinical trials in 2,689 patients reported that the use of a plastic wound protector was associated with a 30% decrease in risk of SSI.^169^
    - There was a significant trend toward greater protective effect using a dual ring protector as compared to a single ring protector: 29% decrease in risk of SSI for dual ring and 16% decrease in risk of SSI for single ring.^169^
    - Another prospective randomized study of dual ring protectors in pancreatectomy showed a reduction in SSI rate from 44% to 21% (*P* = .011) with the use of a dual ring protector.^170^
9. **Perform intraoperative antiseptic wound lavage.**^171^ (Quality of evidence: MODERATE)
  - Wound lavage is a common practice, although the solution and volume used for lavage differs among surgeons.
  - Evidence does not support saline lavage (nonantiseptic lavage) to reduce SSIs.^171,172^
  - Several systematic reviews and meta-analyses support the use of prophylactic intraoperative wound irrigation with sterile dilute povidone-iodine lavage to decrease the risk of SSIs. One systematic review and meta-analysis published in 2017 evaluated 21 RCTs and concluded that lavage with sterile dilute povidone-iodine decreased the risk of SSI compared to nonantiseptic lavage (odds ratio [OR], 0.31; 95% confidence interval [Cl], 0.13–0.73).^102,173^ This study reported no benefit from antibiotic irrigation and discouraged this practice.
  - A systematic review and network meta-analysis published in 2021 reported that relative to saline lavage, both antibiotic irrigation (OR, 0.439; 95% CI, 0.282–0.667) and sterile dilute povidone-iodine (OR, 0.573; 95% CI, 0.321–0.953) decreased the risk of SSI. A third systematic review and meta-analysis published in 2015 reported a similar benefit of antibiotic irrigation and sterile dilute povidone-iodine in the subgroup analysis focused on colorectal surgery.^174,175^ Data were mixed in a different meta-analysis published in 2019,^176^ potentially due to whether the antibiotic lavage (typically a β-lactam or aminoglycoside agent) was used in clean–clean-contaminated or contaminated–dirty wounds.
  - We recommend the use of dilute povidone-iodine lavage over saline lavage, making sure that sterility is maintained during preparation and administration to enhance patient safety. We recommend studying antibiotic irrigation versus dilute povidone-iodine irrigation in an RCT focused on intra-abdominal surgery that is contaminated–dirty.
  - Given the dearth of povidone-iodine solutions formally labeled “sterile,” we advise surgeons to educate themselves as to their options and to carefully weigh the risks and benefits of using povidone-iodine solutions available at their facility.
  - Bacitracin is contraindicated. The FDA withdrew injectable bacitracin from the market because safety concerns outweighed the benefits. This was based on case reports of intraoperative anaphylactic shock associated with bacitracin irrigation.^177^
  - Other agents worth additional study include polyhexanide and rifampicin in certain patient populations.^178,179^
10. **Control blood-glucose level during the immediate postoperative period for all patients.**^94^ (Quality of evidence: HIGH)
  - Monitor and maintain postoperative blood-glucose level regardless of diabetes status.
  - Maintain postoperative blood-glucose level between 110 and 150 mg/dL. Increased glucose levels during the operational procedure are associated with higher levels in the postoperative setting.^180^ Studies on postoperative blood glucose have focused on monitoring through postoperative day 1–2; however, heterogeneity between studies makes it impossible to recommend a definitive window for postoperative blood-glucose control other than 24–48 hours.^94,180-185^
  - The ideal method for maintaining target postoperative blood-glucose level remains unknown. Generally, continuous insulin-infusion protocols lead to better control than subcutaneous insulin (sliding scale) strategies.^186^ Continuous insulin infusion commonly requires intensive monitoring; thus, its use in the ambulatory surgery is often not feasible.
  - Intensive postoperative blood-glucose control (targeting levels <110 mg/dL) has not consistently shown reduced risk of SSI. Although some studies have demonstrated decreased SSI rates,^187^ others have demonstrated higher rates of hypoglycemia and adverse outcomes including stroke and death.^188^
11. **Use a checklist and/or bundle to ensure compliance with best practices to improve surgical patient safety.** (Quality of evidence: HIGH)
  - The World Health Organization (WHO) checklist is a 19-item surgical safety checklist to improve adherence with best practices.^189^
    - A multicenter, quasi-experimental study conducted across 8 countries demonstrated that use of the WHO checklist led to lower surgical complication rates, including SSI and death.^190^
    - These findings have been confirmed in subsequent single- and multicenter quasi-experimental studies.^191,192^
  - Overall, the use of bundles can reduce SSI, but the exact elements needed in a bundle are unknown.^193^ This issue is important because some elements have considerable cost and logistical implications, so it is important to understand the impact of individual elements outside a bundle.^193^
12. **Perform surveillance for SSI.** (Quality of evidence: MODERATE)
  - Identify high-risk, high-volume operative procedures to be targeted for SSI surveillance based on a risk assessment of patient populations, operative procedures performed, and available SSI surveillance data. Some surveillance is also mandated by federal and state regulations.
  - Identify, collect, store, and analyze data needed for the surveillance program.4
    - Develop a database for storing, managing, and accessing data collected on SSIs.
    - Implement a system for collecting data needed to identify and report SSIs. This is discussed in Section 2. Consider collecting data on patient comorbidities (including American Society of Anesthesiology [ASA] score and specific risk factors such as body mass index and diabetes), surgical factors (including wound class, operative duration), process measures (including completion of essential practices discussed in this section), and specifics of SSI (including depth, infecting organism, and antimicrobial susceptibilities).
    - Develop a system for routine review and interpretation of SSI rates and/or SIRs to detect significant increases or outbreaks and to identify areas where additional resources might be needed to improve SSI rates.^34,194^ If increased rates are identified, determine the number of infections that were potentially preventable.^195^
  - Convene key national agencies, organizations, and societies to evaluate. Where possible, align definitions and reporting requirements.
13. **Increase the efficiency of surveillance by utilizing automated data.** (Quality of evidence: MODERATE)
  - Implement a method to electronically transmit data to infection prevention and control personnel needed to determine denominator data and calculate SSI rates for various procedures. This might include procedure data, process measure data, readmission and rehospitalization data, postoperative antimicrobial data, microbiology data, and diagnosis and procedure codes.^54,196-199^
14. **Provide ongoing SSI rate feedback to surgical and perioperative personnel and leadership.** (Quality of evidence: MODERATE)
  - Routinely audit and provide confidential feedback on SSI rates or SIRs and adherence to process measures to individual surgeons, the surgical division and/or department chiefs, and hospital leadership.^4,200^
    - Provide risk-adjusted SSI SIRs for each type of procedure under surveillance and reported to the NHSN. For procedures not reported to the NHSN, there may be alternative data to review through surveillance programs such as National Surgical Quality Improvement Program (NSQIP).^201^
    - Anonymously benchmark procedure-specific, risk-adjusted SSI SIRs among peer surgeons.
15. **Measure and provide feedback to HCP regarding rates of compliance with process measures.**^94^ (Quality of evidence: LOW)
  - Routinely provide feedback to surgical staff, perioperative personnel, and leadership regarding compliance with targeted process measures.^195^
16. **Educate surgeons and perioperative personnel about SSI prevention measures.** (Quality of evidence: LOW)
  - Include risk factors, outcomes associated with SSI, local epidemiology (eg, SSI rates by procedure, rate of methicillin-resistant *Staphylococcus aureus* [MRSA] infection in a facility), and essential prevention measures.
17. **Educate patients and their families about SSI prevention as appropriate.** (Quality of evidence: LOW)
  - Provide instructions and information to patients prior to surgery describing strategies for reducing SSI risk. Specifically provide preprinted materials to patients.^202^
  - Examples of printed materials for patients are available from the following web pages:
    - *JAMA* patient page: Wound Infections^87^
    - Surgical Care Improvement Project Tips for Safer Surgery^203^
    - CDC Frequently Asked Questions About Surgical-Site Infections^204^
    - SHEA Infection Prevention Handout for Patients and Visitors^205^
18. **Implement policies and practices to reduce the risk of SSI for patients that align with applicable evidence-based standards, rules and regulations, and medical device manufacturer instructions for use.**^4,94^ (Quality of evidence: MODERATE)
  - Implement policies and practices to reduce modifiable risk factors (Table 1), including the following:
    - Optimally disinfect the hands of the surgical team members.
    - Adhere to hand hygiene practices, including nonsurgeon members of the operating team.^206^
    - Reduce unnecessary traffic in operating rooms.^207,208^
    - Avoid use of nonsterile water sources in the operating room.^209,210^
    - Properly care for and maintain the operating rooms, including appropriate air handling, pressure relative to hallway, temperature, humidity, and optimal cleaning and disinfection of equipment and the environment.^4^
    - Maintain asepsis from the start of preparation of surgical instruments on the sterile field through wound closure and dressing.
    - Establish a robust infection control risk assessment program focused on mitigating risk during construction projects.
    - Proactively address potential risks from supply-chain shortages and communicate to frontline teams.
    - Discuss any staffing shortages and potential impact on outcomes as they relate to compliance with SSI prevention measures.
19. **Observe and review operating-room personnel and the environment of care in the operating room and in central sterile reprocessing.** (Quality of evidence: LOW)
  - Perform direct observation audits of operating-room personnel to assess operating-room processes and practices to identify infection control lapses, including but not limited to adherence to process measures (antimicrobial prophylaxis choice, timing and duration protocols, hair removal, etc), surgical hand antisepsis, patient skin preparation, operative technique, surgical attire (wearing and/or laundering outside the operating room), and level of operating-room traffic.^211-215^ Perform remediation when breaches of standards are identified.
    - Operating-room personnel should include surgeons, surgical technologists, anesthesiologists, circulating nurses, residents, medical students, trainees, and device manufacturer representatives.^211^
  - Perform direct observation audits of environmental cleaning practices in the operating room, instrument reprocessing (sterilization) area, and storage facilities.
    - Review instrument reprocessing and flash sterilization or immediate-use steam sterilization (IUSS) logs.
    - Review maintenance records for operating room heating, ventilation, and air conditioning (HVAC) system including, results of temperature, relative humidify, and positive air pressure maintenance testing in the operating rooms(s).
  - Provide feedback and review infection control measures with operating-room and environmental personnel.

### Additional approaches for preventing SSI

These additional approaches can be considered when hospitals have successfully implemented essential practices and seek to further improve outcomes in specific locations and/or patient populations.

1. **Perform an SSI risk assessment.** (Qualify of Evidence: LOW)
  - Convene a multidisciplinary team (eg, surgical leadership, hospital administration, qualify management services, and infection control) to identify gaps, improve performance, measure compliance, assess impacts of interventions, and provide feedback.^216^
2. **Consider use of negative-pressure dressings in patients who may benefit.** (Quality of Evidence: MODERATE)
  - Negative-pressure dressings placed over closed incisions are thought to work by reducing fluid accumulation in the wound. Recent systematic reviews have demonstrated a significant reduction in SSI with their use.^217-219^
  - These dressings have been particularly noted to reduce SSIs in patients who have undergone abdominal surgery^220,221^ and joint arthroplasty,^222,223^ although not all studies have shown benefit^224^ and some indicate benefit only in a subset of procedures such as revision arthroplasty.^222^
  - Guidance is lacking regarding which patients most benefit from the use of negative-pres sure dressings, with some evidence that the benefit increases with age and body mass index.^225^
  - Negative-pressure dressings seem most successful at reducing superficial SSIs,^226^ but some risk of blistering has been observed.^222^ These blisters could lead to breaks in the skin that might increase risk of infection.
  - It is important to assess the ability of the patient to manage a negative-pressure dressing, particularly if used in the ambulatory setting.
  - Cost-effectiveness studies of negative-pressure dressings are needed.
3. **Observe and review practices in the preoperative clinic, postanesthesia care unit, surgical intensive care unit, and/or surgical ward.** (Quality of evidence: MODERATE)
  - Perform direct observation audits of hand-hygiene practices among all HCP with direct patient contact.^213^
  - Evaluate wound care practices.^227^
  - Perform direct observation audits of environmental cleaning practices.
  - Provide feedback and review infection control measures with HCP in these perioperative care settings.
4. **Use antiseptic-impregnated sutures as a strategy to prevent SSI.** (Quality of evidence: MODERATE)
  - Human volunteer studies involving foreign bodies have demonstrated that the presence of surgical sutures decreases the inoculum required to cause an SSI from 10^6^ to 10^2^ organisms.^228^
  - Some trials have shown that surgical wound closure with triclosan-coated polyglactin 910 antimicrobial sutures may decrease the risk of SSI compared to standard sutures.^229,230^ For example, an RCT of 410 colorectal surgeries concluded that the rate of SSI decreased >50% among patients who received antimicrobial sutures (9.3% in control group vs 4.3 among cases; *P* = .05).^231^
  - In contrast, a systematic review and meta-analysis evaluated 7 RCTs and concluded that neither SSI rates (OR, 0.77; 95% CI, 0.4–1.51; *P* = .45) nor wound dehiscence rates (OR, 1.07; 95% CI, 0.21–5.43; *P* = .93) were statistically different compared to controls.^232^ In addition, a small study raised concern about higher wound dehiscence rates associated with using these antimicrobial sutures.^233^
  - The impact of routinely using antiseptic-impregnated sutures on the development of antiseptic resistance remains unknown.

### Approaches that should not be considered a routine part of SSI prevention

1. **Do not routinely use vancomycin for antimicrobial prophylaxis.**^73^ (Quality of evidence: MODERATE)
  - Vancomycin should not routinely be used for antimicrobial prophylaxis, but it can be an appropriate agent for specific scenarios.^128,234^ Reserve vancomycin for specific clinical circumstances, as in patients who are known to be MRSA colonized (including those identified on preoperative screening), particularly if the surgery involves prosthetic material. Vancomycin can also be used in the setting of a proven outbreak of SSIs due to MRSA.^235^
    - Suspected high rates of MRSA SSI should not be used as justification for vancomycin use. In a cohort study of 79,092 surgical procedures, the primary reason for vancomycin perioperative prophylaxis was the perception of high facility rates of MRSA or high-risk procedure for MRSA. Patients who received vancomycin prophylaxis because of the perceived high facility risk of MRSA had no increase in prevalence of MRSA colonization compared with the general surgical population. The incidence of SSIs was the same regardless of vancomycin prophylaxis, but the incidence of acute kidney injury (AKI) was significantly higher among patients who received vancomycin.^236^
    - In a retrospective cohort study of 79,058 surgical procedures, vancomycin perioperative prophylaxis was inde-pendently associated with significantly increased risk of AKI.^107^
    - Two meta-analyses of studies comparing glycopeptides to β-lactam antimicrobial prophylaxis concluded that there was no difference in rates of SSI between the 2 antimicrobial prophylaxis regimens.^125,237^
  - Vancomycin does not have activity against gram-negative pathogens and appears to have less activity against MSSA than β-lactam agents. The addition of vancomycin to standard antimicrobial prophylaxis has been done in specific circumstances, but the benefits should be weighed against the risks.^73,237-239^
    - Among cardiac surgery patients, receipt of vancomycin in combination with a β-lactam for perioperative prophylaxis was associated with increased AKI compared with either antibiotic alone^107,240^
    - In a cohort study of 70,101 surgical cases, vancomycin plus β-lactam combination prophylaxis was associated with a greater risk of AKI compared with vancomycin alone.^241^ In that study, vancomycin plus a β-lactam reduced the incidence of SSIs following cardiothoracic procedures compared with either antibiotic alone. However, this antimicrobial combination did not reduce SSIs for orthopedic, vascular, hysterectomy, or colorectal procedures.
2. **Do not routinely delay surgery to provide parenteral nutrition.** (Quality of evidence: HIGH)
  - Preoperative administration of total parenteral nutrition (TPN) has not been shown to reduce the risk of SSI in prospective RCTs and may increase the risk of SSI.^242,243^
  - Individual trials comparing enteral and parenteral perioperative nutrition and comparing immunomodulating diets containing arginine and/or glutamine to standard control diets tend to have very small sample sizes and fail to show significant differences in SSI rates. In 2 recent meta-analyses, however, postoperative infectious complications were reduced in patients receiving enteral diets containing glutamine and/or arginine administered either before or after the surgical procedure.^244,245^
3. **Do not routinely use antiseptic drapes as a strategy to prevent SSI.** (Quality of evidence: HIGH)
  - An incise drape is an adhesive film that covers the surgical incision site to minimize bacterial wound contamination from endogenous flora. These drapes can be impregnated with antiseptic chemicals such as iodophors.
    - A 2007 Cochrane review of 5 trials concluded, nonantiseptic incise drapes were associated with a higher risk of SSIs compared to no incise drapes (RR, 1.23; 95% CI, 1.02–1.48)^246^ although this association may have been heavily weighted by one specific study.^247^
    - Two trials (abdominal and cardiac surgical patients) compared iodophor-impregnated drapes to no drapes.^247,248^ Although wound contamination was decreased in one trial,^247^ neither trial demonstrated that iodophor-impregnated drapes decreased the rate of SSI.
    - A nonrandomized retrospective study similarly concluded that impregnated drapes do not prevent SSI after hernia repair.^249^

### Unresolved issues

1. **Optimize tissue oxygenation at the incision site.**
  - In a meta-analysis of 5 studies, perioperative supplemental oxygen administration led to a relative SSI risk reduction of 25%. In contrast, a more recent meta-analysis of 15 studies was inconclusive.^250^ Additional studies published since the 2014 SHEA Compendium have similarly not shown a reduction in SSI in patients who received supplemental oxygen at a fraction of inspired oxygen (FiO_2_) of 80%.^251-253^
  - Most trials compared 80% FiO_2_ to 20%–35% FiO_2_. The benefit of other oxygen concentrations remains unknown.
  - The best available evidence for the use of supplemental oxygen is in patients undergoing high-risk surgery with general anesthesia using mechanical ventilation.^254-256^
  - Supplemental oxygen is most effective when combined with additional strategies to improve tissue oxygenation including maintenance of normothermia and appropriate volume replacement. Tissue oxygenation at the incision site depends on vasoconstriction, temperature, blood supply, and cardiac output.
2. **Preoperative intranasal and pharyngeal CHG treatment for patients undergoing cardiothoracic procedures**
  - Although data from an RCT trial support the use of CHG nasal cream combined with 0.12% CHG mouthwash,^257^ CHG nasal cream is neither FDA approved nor commercially available in the United States.
3. **Use of gentamicin-collagen sponges**
  - Gentamicin-collagen sponges have been evaluated as an intervention to decrease SSI among colorectal and cardiac surgical patients.
    - Colorectal surgical patients. Several single-center randomized trials demonstrated that gentamicin-collagen sponges decrease the risk of SSI following colorectal procedures.^258-260^ However, the rate of SSI was higher with the sponge in 2 recent, large, multicenter RCTs.^261,262^
    - Cardiothoracic surgical patients. Four RCTs have evaluated the use of gentamicin-collagen sponges in cardiothoracic surgery. Three of these trials demonstrated a decrease in SSIs and one demonstrated no difference.^263-266^ A recent meta-analysis combining these trials and 10 observational studies concluded that the risk of deep sternal wound infection was significantly lower in patients who received a gentamicin-collagen sponge than patients who did not (RR, 0.61; 95% CI, 0.39–0.98) despite significant heterogeneity among the trials.^267^
  - Gentamicin-collagen sponges are not currently FDA approved for use in the United States.
4. **Use of antimicrobial powder**
  - Multiple publications have examined the use of vancomycin powder in surgical incisions, especially for spinal and cranial procedures for which *S. aureus* is a primary pathogen.^268,269^ Although a few reviews report a lower rate of SSI in spinal surgery with the use of vancomycin powder,^270^ other references report a significant increase in the proportion of SSI with polymicrobial and gram-negative pathogens when they occur.^271-273^ In addition, a prospective randomized trial comparing the use of vancomycin powder in combination with intravenous vancomycin to the use of intravenous vancomycin alone found no benefit with the addition of vancomycin powder.^274^
5. **Use of surgical attire**
  - Although there are longstanding traditions and opinions regarding surgical attire in the operating room, no strong evidence exists for many of them. It has not been demonstrated that surgical attire affects SSI rates.^275^ One approach to managing issues pertaining to surgical attire is to form a multidisciplinary body including infection control, surgery, nursing, and anesthesia to discuss and agree to some sensible, not overly aggressive or cumbersome attire standards, and to establish policies and procedures that are compliant with state and CMS requirements.^275^

## Section 5: Performance measures

### Internal reporting

These performance measures are intended to support internal hospital quality improvement efforts and do not necessarily address external reporting needs. The process and outcome measures suggested here are derived from published guidelines, other relevant literature, and the opinion of the authors. Report process and outcome measures to senior hospital leadership, nursing leadership, and clinicians who care for patients at risk for SSI (Table 4).

### Process measures

EXAMPLE: Compliance with antimicrobial prophylaxis guidelines

1. Measure the percentage of procedures in which antimicrobial prophylaxis was provided appropriately. Appropriateness includes (1) correct antibiotic for specific surgery, (2) correct antibiotic dose, (3) administration start time within 1 hour of incision (2 hours allowed for vancomycin and fluoroquinolones), and (4) discontinuation of the agent after skin closure.
  - Numerator: Number of patients who appropriately received antimicrobial prophylaxis.
  - Denominator: Total number of selected operations performed.
  - Multiply by 100 so that measure is expressed as a percentage.

### Outcome measures

EXAMPLE: Surgical site infection SIR

1. Use NHSN definitions and risk adjustment methods for measuring SSI incidence^43^
  - SIR numerator: Number of surgical site infections following a specified type of procedure.
  - SIR denominator: Total number of predicted SSIs following a specified type of procedure. The SIR denominator is calculated in NHSN using national baseline data and is risk adjusted for several facility, patient, and procedure-level factors.^34^
  - SIR is the ratio of the observed (O) number of SSIs that occurred compared to the predicted (P) number for a specific type of procedure: SIR = O/P.^34^ Values that exceed 1.0 indicate that more SSIs occurred than expected. Importantly, SIR can only be calculated if the number of predicted HAIs is ≥1. Thus, this approach maybe more difficult for small surgical programs or if few procedures are performed for any 1 procedure type.^276^
  - Risk adjustment using logistic regression and the SIR method generally provides better risk adjustment than the traditional NHSN risk index.^281,285^

### External reporting

There are many challenges in providing useful information to consumers and other working partners while preventing unintended consequences of public reporting of HAIs.^283-285^ Recommendations and requirements for public reporting of HAIs have been provided by HICPAC,^286,287^ the National Quality Forum,^288^ and the CMS^289^ (Table 5).

### Outcome measures

1. External reporting measures now focus mostly on outcomes.
2. Since 2012, the CMS has imposed a reporting requirement for SSI data for inpatient abdominal hysterectomy and inpatient colon procedures.^290,291^
3. Federal and state requirements
  - Federal requirements
    - CMS published a final rule in the *Federal Register* on August 18, 2011 that includes surgical site infection (SSI) reporting via the NHSN in the CMS Hospital Inpatient Quality Reporting (IQR) Program requirements for 2012.^289^ More specifically, the rule announced a reporting requirement for SSI data for inpatient abdominal hysterectomy and inpatient colon procedures.^291^
    - The requirements for SSI reporting to the NHSN for the hospital IQR program do not preempt or supersede state mandates for SSI reporting to NHSN (ie, hospitals in states with a SSI reporting mandate must abide by their state’s requirements, even if they are more extensive than the requirements for this CMS program). NHSN users reporting SSI data to the system must adhere to the definitions and reporting requirements for SSIs as specified in the NHSN Patient Safety Component Protocol Manual.^43,291^
  - State requirements. Hospitals in states that have mandatory SSI reporting requirements must collect and report the data required by the state. For information on state requirements, check with your state or local health department.

### External quality initiatives

Several external quality initiatives focused on SSI prevention are ongoing. The benefits from participation in these external quality initiatives are unknown but may include improvement in the culture of safety and patient outcomes, including decreased rates of SSI.^292^

## Section 6: Implementation of SSI prevention strategies

SSI prevention science and education must be partnered with purposeful implementation of interventions to achieve desired outcomes. Beyond protocol development and educational efforts, this includes measurement of adherence to agreed-upon practices, understanding and addressing potential barriers to adherence, and frequent feedback to all partners.

Reliability is the frequency at which an intervention is completed when indicated. Implementation of any practice requires monitoring for reliability, commonly known as a process measure. In SSIs, process measurement is especially important to successful implementation due to the complexity of systems involved and of the outcome itself. Connecting a reduction or increase in SSI rates to utilization of a bundle is difficult without reliability measurement, and protocol adherence has been directly correlated to improved outcomes.^293^ Successful implementation efforts described in the literature have frequently failed to identify a single effective intervention, instead emphasizing the effect of process reliability.^294-296^

High reliability can be achieved through different methods and conceptual frameworks. The following outline summarizes ways in which facilities have achieved reliability. Choice of a method for a given group depends on system context,^297,298^ local knowledge of improvement and implementation science, and resources available to support the effort.

1. Quality improvement tools
  - Team projects. Implementation often occurs in the context of a team project, such as that used to teach and disseminate quality improvement methods. Utilizing a planned quality improvement project may be a good approach for initial implementation of an existing or novel bundled intervention.^299-302^ Because SSIs may present weeks to months after surgery and because new systems need time to adjust, SSI prevention implementation may take longer than the typical 90–120 days of a quality improvement project and may benefit from an iterative and adaptive approach over time.^303^
  - Process mapping. Understanding the system involved may help in planning more effective interventions, particularly in resource-constrained settings.^304^
  - Reliability measurement. Process reliability should be measured regularly. SSI prevention process measures like antibiotic choice or timing of administration of preoperative antibiotics may be measurable using existing data available in an electronic health record.^305^ Other behaviors, such as environmental cleaning practices, may require direct observation.^306^
  - Feedback. Sharing results with working partners is an important way to change and solidify behavior. Increasing awareness among HCP throughout the surgical care continuum,^31,307-310^ including sharing outcome data with individual surgeons, has been effective in a variety of contexts.^308,311^
  - Apparent cause analysis. Learning from failed processes or unwanted outcomes is a useful means to gain a shared mental model and advance efforts. Objective review of data helps avoid assigning blame to individuals and focusing on needed system improvements.
  - Surveillance and improvement networks. Networks of institutions within the US and internationally have arisen to collect data, learn collectively, and improve patient outcomes.^312,313^ Groups such as Solutions for Patient Safety,^314^ the NSQIP,^315^ and statewide collaboratives^316^ have helped facilitate improvement through direct engagement or supplying data to drive interventions. Punitive approaches have been less effective at affecting improvement.^283^
2. Multidisciplinary approach (Table 6)
  - Efforts to prevent SSIs should consider the large variety of touch points, risk factors, and partners needed to implement multiple effective strategies.^31,295,296,317-319^ Partners from all areas should be included in the prevention effort, such as preoperative clinic staff, perioperative staff, staff in sterile processing, postoperative staff, pharmacists, etc.
  - Frontline involvement. SSI prevention is not the sole responsibility of surgeons and involves mitigating risk inside and outside operating rooms. Recruiting nonsurgeon groups, such as medical or nursing trainees or pharmacists^320^ to lead improvement efforts, has been shown to be effective.
  - Education and reinforcement. Orienting patients, families, and care providers to the need to prevent SSI by implementing interventions pre-, intra-, and postoperatively is crucial. Emphasizing interventions that they can control has been effective at reducing SSIs.^31,202,321-324^ Education should be provided to patients and families in their primary languages.
3. Human factors engineering
  - Interventions that automate reminders (eg, alarms to prevent excessive door opening or electronic alerts to re-dose antibiotics)^325,326^ or processes themselves may be effective at preventing SSIs.^325,327^ Existing information systems, such as electronic health records, can be leveraged for this purpose as well as for standardizing evidence-based order sets.
  - Operating-room door openings are a surrogate marker for poor operating-room discipline.^208,327,329^ Agreeing on a limit for how many door openings during surgery are acceptable and staying below that limit have been associated with decreased incidence of SSIs.^328^ Communication between the surgeon and operating-room staff on the equipment needed prior to surgery can lead to fewer door openings.^328^ Operating-room personnel turnover during procedures has been associated with an increased risk of SSI, even after statistically adjusting for length of surgery.^330^ When possible, shift changes and breaks should wait until the procedure has ended.
  - Standardizing practices through the use of dedicated teams, checklists, and surgeon preference cards, and ensuring adequate staffing have all been effective strategies to implement interventions.^31,208,331-333^
  - Interventions to prevent SSIs can be optimized by identifying the people (eg, preoperative nurse, operating room nurse, surgeon, patient, or family) needed to successfully implement the intervention and provide them with directed tools to support adherence with the intervention. The perspectives of each of these partners need to be considered to identify barriers and facilitators to intervention adherence.^334^
4. Accountability
  - Accountability is an essential principle for preventing HAIs by ensuring evidence-based implementation strategies are used consistently, maximizing their effectiveness in preventing HAIs.
  - Engagement and commitment of executive and senior leadership are essential to setting goals, removing barriers, and justifying the effort to build and sustain improvements.^319,335-337^ Engaged local leaders (eg, a senior surgeon) also give the effort and expectations legitimacy.
  - Interventions, bundle components, and practices should be evidence-based as much as possible^338^ and should be deemed appropriate for the surgical population (eg, evidence from the adult population may not be appropriate to apply in a pediatric population).
5. Safety culture and practices
  - SSI prevention efforts align well with, and may be contextualized within, patient and employee safety campaigns. However, culture change is a prolonged and ongoing process. SSI prevention should not be delayed until safety culture is improved, but rather used as a concrete example of the benefits of safe behaviors.

## Figures and Tables

**Table 1.** Summary of Recommendations to Prevent Surgical Site Infections (SSIs)

Essential practices
1. Administer antimicrobial prophylaxis according to evidence-based standards and guidelines.^73,75^ (Quality of evidence: HIGH)
2. Use a combination of parenteral and oral antimicrobial prophylaxis prior to elective colorectal surgery to reduce the risk of SSI.^115,116^ (Quality of evidence: HIGH)
3. Decolonize surgical patients with an anti-staphylococcal agent in the preoperative setting for orthopedic and cardiothoracic procedures. (Quality of evidence: HIGH) Decolonize surgical patients in other procedures at high risk of staphylococcal SSI, such as those involving prosthetic material. (Quality of evidence: LOW)
4. Use antiseptic-containing preoperative vaginal preparation agents for patients undergoing cesarean delivery or hysterectomy. (Quality of evidence: MODERATE)
5. Do not remove hair at the operative site unless the presence of hair will interfere with the surgical procedure.^4,119^ (Quality of evidence: MODERATE)
6. Use alcohol-containing preoperative skin preparatory agents in combination with an antiseptic. (Quality of evidence: HIGH)
7. For procedures not requiring hypothermia, maintain normothermia (temperature > 35.5°C) during the perioperative period. (Quality of evidence: HIGH)
8. Use impervious plastic wound protectors for gastrointestinal and biliary tract surgery. (Quality of evidence: HIGH)
9. Perform intraoperative antiseptic wound lavage.^171^ (Quality of evidence: MODERATE)
10. Control blood-glucose level during the immediate postoperative period for all patients.^94^ (Quality of evidence: HIGH)
11. Use a checklist and/or bundle to ensure compliance with best practices to improve surgical patient safety. (Quality of evidence: HIGH)
12. Perform surveillance for SSI. (Quality of evidence: MODERATE)
13. Increase the efficiency of surveillance by utilizing automated data. (Quality of evidence: MODERATE)
14. Provide ongoing SSI rate feedback to surgical and perioperative personnel and leadership. (Quality of evidence: MODERATE).
15. Measure and provide feedback to HCP regarding rates of compliance with process measures.^94^ (Quality of evidence: LOW)
16. Educate surgeons and perioperative personnel about SSI prevention measures. (Quality of evidence: LOW)
17. Educate patients and their families about SSI prevention as appropriate. (Quality of evidence: LOW)
18. Implement policies and practices to reduce the risk of SSI for patients that align with applicable evidence-based standards, rules and regulations, and medical device manufacturer instructions for use.^4,94^ (Quality of evidence: MODERATE)
19. Observe and review operating room personnel and the environment of care in the operating room and in central sterile reprocessing. (Quality of evidence: LOW)
Additional approaches
1. Perform an SSI risk assessment. (Quality of evidence: LOW)
2. Consider use of negative pressure dressings in patients who may benefit. (Quality of evidence: MODERATE)
3. Observe and review practices in the preoperative clinic, postanesthesia care unit, surgical intensive care unit and/or surgical ward. (Quality of evidence: MODERATE)
4. Use antiseptic-impregnated sutures as a strategy to prevent SSL (Quality of evidence: MODERATE)
Approaches that should not be considered a routine part of SSI prevention
1. Do not routinely use vancomycin for antimicrobial prophylaxis.^73^ (Quality of evidence: MODERATE)
2. Do not routinely delay surgery to provide parenteral nutrition. (Quality of evidence: HIGH)
3. Do not routinely use antiseptic drapes as a strategy to prevent SSL (Quality of evidence: HIGH)
Unresolved issues
1. Optimize tissue oxygenation at the incision site
2. Preoperative intranasal and pharyngeal CHG treatment for patients undergoing cardiothoracic procedures
3. Use of gentamicin-collagen sponges
4. Use of antimicrobial powder
5. Use of surgical attire

**Table 2.** Quality of Evidence^a^

HIGH	Highly confident that the true effect lies close to that of the estimated size and direction of the effect, for example, when there are a wide range of studies with no major limitations, there is little variation between studies, and the summary estimate has a narrow confidence interval.
MODERATE	The true effect is likely to be close to the estimated size and direction of the effect, but there is a possibility that it is substantially different, for example, when there are only a few studies and some have limitations but not major flaws, there is some variation between studies, or the confidence interval of the summary estimate is wide.
LOW	The true effect may be substantially different from the estimated size and direction of the effect, for example, when supporting studies have major flaws, there is important variation between studies, the confidence interval of the summary estimate is very wide, or there are no rigorous studies.

a Based on the CDC Healthcare Infection Control Practices Advisory Committee (HICPAC) “Update to the Centers for Disease Control and Prevention and the Healthcare Infection Control Practices Advisory Committee Recommendations Categorization Scheme for Infection Control and Prevention Guideline Recommendations” (October 2019), the Grades of Recommendation, Assessment, Development, and Evaluation (GRADE),^339^ and the Canadian Task Force on Preventive Health Care.^340^

**Table 3.** Selected Risk Factors for and Recommendations to Prevent Surgical Site Infection (SSI)

Risk Factor	Recommendation	Quality of Evidence
***Intrinsic, patient-related (preoperative)***		
**Unmodifiable**		
Age	No formal recommendation: relationship to increased risk of SSI may be secondary to comorbidities or immunosenescence^341-343^	N/A
History of radiation	No formal recommendation. Prior irradiation at the surgical site increases the risk of SSI, likely due to tissue damage and wound ischemia.^183^	N/A
History of skin and soft-tissue infections	No formal recommendation. History of a prior skin infection may be a marker for inherent differences in host immune function.^344^	N/A
**Modifiable**		
Glucose control	Control serum blood-glucose levels for all surgical patients including patients without diabetes.^345^	HIGH
Obesity	Increase dosing of prophylactic antimicrobial agent for morbidly obese patients.^73,346^	HIGH
Smoking cessation	Encourage smoking cessation within 30 days of procedure.^4,347-351^	HIGH
Immunosuppressive medications	Avoid immune-suppressive medications in perioperative period if possible	LOW
Hypoalbuminemia	No formal recommendation. Though a noted risk factor,^352^ do not delay surgery for use of total parenteral nutrition.	N/A
*S. aureus* nasal colonization	Decolonize patients with nasal mupirocin or povidine-iodine prior to surgery	MODERATE
***Preparation of patient***		
Hair removal	Do not remove unless hair will interfere with the operation^4^; if hair removal is necessary, remove outside of the operating room by clipping. Do not use razors.	HIGH
Preoperative infections	Identify and treat infections remote to the surgical site (eg, urinary tract infection in the presence of prior to elective surgery.^4,353^ Do not routinely test or treat for asymptomatic bacteriuria except in urologic procedures.^4,353^	MODERATE
***Operative characteristics***		
Surgical scrub (surgical team members’ hands and forearms)	Use appropriate antiseptic agent to perform preoperative surgical scrub.^4,354^ For most products, scrub the hands and forearms for 2–5 minutes.	MODERATE
Skin preparation	Wash and clean skin around incision site. Use a dual agent skin prep containing alcohol unless contraindications exist.^4^	HIGH
Antimicrobial prophylaxis	Administer only when indicated.^4^ Select appropriate agents based on surgical procedure, most common pathogens causing SSI for a specific procedure, and published recommendations.^73^ Administer within 1 hour of incision to maximize tissue concentration.^73^ Discontinue antimicrobial agents after incisional closure in the operating room.^a^	HIGH
Blood transfusion	Blood transfusions increase the risk of SSI by decreasing macrophage function. Reduce blood loss and need for blood transfusion to greatest extent possible.^355-357^	MODERATE
Surgeon skill/technique	Handle tissue carefully and eradicate dead space.^4^	LOW
Appropriate gloving	All members of the operative team should double glove and change gloves when perforation is noted.^358^	LOW
Asepsis	Adhere to standard principles of operating room asepsis.^4^	LOW
Operative time	No formal recommendation in most recent guidelines; minimize as much as possible without sacrificing surgical technique and aseptic practice.	HIGH
***Operating room characteristics***		
Ventilation	Follow American Institute of Architects’ recommendations for proper air handling in the operating room.^4,359^	LOW
Traffic	Minimize operating room traffic.^4,207,208^	LOW
Environmental surfaces	Use an Environmental Protection Agency (EPA)–approved hospital disinfectant to clean visibly soiled or contaminated surfaces and equipment in accordance with manufacturer’s instructions.^4^	LOW
Sterilization of surgical equipment	Sterilize all surgical equipment according the device manufacturer’s validated parameters: cycle type, time, temperature, pressure, and dry time. Minimize the use of immediate use steam sterilization.^4,360^	MODERATE

a Vancomycin and fluoroquinolones can be given 2 hours prior to incision.

**Table 4.** SSI Prevention Internal Reporting Process and Outcome Measures

Internal Reporting Process Measure Example: Compliance with Antimicrobial Prophylaxis Guidelines
Percentage of procedures in which antimicrobial prophylaxis was provided appropriately = (No. of patients who appropriately received antimicrobial prophylaxis/Total number of selected operations performed) ×100 1. Correct antibiotic for specific surgery 2. Correct antibiotic dose 3. Administrative start time within 1 hour of incision (2 hours allowed for vancomycin and fluroquinolones) 4. Discontinuation of agent after skin closure
Internal Reporting Outcome Measure Example: Surgical Site Infection Standardized Infection Ratio (SIR)
SIR = Ratio of observed number of SSIs (O)/Predicted number of SSIs (P) for a specific type of procedure^278^

**Table 5.** SSI Prevention External Reporting Outcome Measures

Federal requirements^a^
1. Reported via CDC NHSN in the CMS Hospital Inpatient Quality Reporting program.^289^ 2. Since 2012, SSI data reporting for inpatient abdominal hysterectomy and inpatient colon procedures has been required.^290,291^ 3. Hospitals in states with a SSI reporting mandate must abide by their state’s requirements, even if they are more extensive than federal requirements.
State requirements and collaboratives
1. In states with mandatory SSI reporting requirements, hospitals must collect and report the data required by the state. 2. Hospitals should check with the state or local health department for requirements.

Note. CDC, Centers for Disease Control and Prevention; NHSN, National Health Safety Network. CMS, Centers for Medicare & Medicaid Services; HICPAC, Healthcare Infection Control Practices Advisory Committee.

a Recommendations and requirements for public reporting provided by HICPAC,^286,287^ the National Quality Forum,^288^ and the CMS.^289^

**Table 6.** Fundamental Elements of Accountability and Engagement for SSI Prevention

Organizational Role	Responsibilities	Accountability
Senior management (executives, senior directors) (Note: regulatory requirement for US hospitals)	Ensure sufficient funds, expertise, and commitment to an infection prevention and control (IPC) program that effectively prevents healthcare-associated infections (HAIs) and the transmission of epidemiologically important pathogens.	Accountable for proper resource allocation and evaluation, including training, competency, and ancillary support (eg, data analysis).
Surgical services leadership (surgeon, anesthesia, perioperative nursing leaders)	Ensure all perioperative staff are aware of their roles and expectations as they relate to SSI prevention. Advocate for the support of senior leadership.	Direct evaluation of groups and practitioners, enforcing standards and correcting when necessary. Review of longitudinal outcome data and communication with all perioperative staff.
Surgical services staff (surgeons, anesthesiologists/CRNAs, perioperative nurses and technicians)	Ensure execution of prevention measures consistently for all procedures. Escalate questions and concerns to senior surgical leadership.	SSI prevention process measurement, individual reinforcement, support, and correction as indicated.
Pharmacists	Ensure proper medications for SSI prevention are available when needed. Promote evidence-based, cost-effective choice of antimicrobial prophylaxis.	Track utilization patterns and adverse drug events to ensure proper use of drugs for SSI prevention. Communicate changes and their rationale (eg, drug shortage, new evidence)
Infection preventionists	Ensure surveillance for SSI is thorough and aligns with national standards. Support prevention efforts as subject-matter experts, coaches, and observers of process and outcome. Educate staff and audit compliance on practical application^361^ of infection control related policies and processes	Validation of surveillance methodology with transparency to all partners. Assess SSI prevention system as a whole to identify gaps and opportunities.
Environmental services staff	Ensure correct processes for cleaning perioperative and related areas, and adequate number, training, and support of staff.	Track benchmarks and conduct process and performance reviews regularly.
Information services	Support SSI prevention efforts through data collection automation and analysis, leverage different platforms (electronic health record, billing databases) to ensure standard and consistent data streams.	Validate systems regularly and whenever updated, maintain flexibility for changes as needs evolve. Engage with other partners if changes are anticipated. Communicate changes to all partners.
